# The Influence of Gender and Self-Efficacy on Healthy Eating in a Low-Income Urban Population Affected by Structural Changes to the Food Environment

**DOI:** 10.1155/2014/908391

**Published:** 2014-03-27

**Authors:** Brenda Robles, Lisa V. Smith, Mirna Ponce, Jennifer Piron, Tony Kuo

**Affiliations:** ^1^Division of Chronic Disease and Injury Prevention, Los Angeles County Department of Public Health, 3530 Wilshire Boulevard, 8th Floor, Los Angeles, CA 90010, USA; ^2^Office of Health Assessment and Epidemiology, Los Angeles County Department of Public Health, 313 North Figueroa Street, Room 127, Los Angeles, CA 90012, USA; ^3^Department of Epidemiology, UCLA Jonathan and Karin Fielding School of Public Health, Box 951772, 71-254 CHS, Los Angeles, CA 90095-1772, USA; ^4^Department of Family Medicine, David Geffen School of Medicine at UCLA, 10880 Wilshire Boulevard, Suite 1800, Los Angeles, CA 90024-4142, USA

## Abstract

Although US obesity prevention efforts have begun to implement a variety of system and environmental change strategies to address the underlying socioecological barriers to healthy eating, factors which can impede or facilitate community acceptance of such interventions are often poorly understood. This is due, in part, to the paucity of subpopulation health data that are available to help guide local planning and decision-making. We contribute to this gap in practice by examining area-specific health data for a population targeted by federally funded nutrition interventions in Los Angeles County. Using data from a local health assessment that collected information on sociodemographics, self-reported health behaviors, and objectively measured height, weight, and blood pressure for a subset of low-income adults (*n* = 720), we compared health risks and predictors of healthy eating across at-risk groups using multivariable modeling analyses. Our main findings indicate being a woman and having high self-efficacy in reading Nutrition Facts labels were strong predictors of healthy eating (*P* < 0.05). These findings suggest that intervening with women may help increase the reach of these nutrition interventions, and that improving self-efficacy in healthy eating through public education and/or by other means can help prime at-risk groups to accept and take advantage of these food environment changes.

## 1. Introduction

In Los Angeles County (~9.8 million residents), health disparities are striking among economically disadvantaged communities [1]. Obesity prevalence is highest among cities with the greatest indices of economic hardship (Table 1). East Compton, for example, has one of the highest rates in the county (39.9%, the city is economically ranked last out of 127 communities), while the city of San Marino has one of the lowest (8.4%, economically ranked first). These marked disparities are observed by race and ethnicity as well, with obesity being more pronounced among Latinos (29.4%) and African Americans (29.2%) [1, 2]. Collectively, this community snapshot paints a picture of significant health disparities in the region [3, 4].

In the literature, factors such as demographics, geography, culture, community resiliency, and access to affordable, healthy foods have been found to be important mediators of obesity risk [5, 6]. Emerging evidence suggests that, to reduce this risk, interdisciplinary interventions—especially in nutrition—should be implemented across multiple sectors (e.g., healthcare, public health, education, transportation, and food environments) [5–7]. In applying this evidence, federal and local health authorities have begun to take notable actions; that is, many recent federally funded obesity prevention efforts have employed an array of practice-based system and environmental (SE) change strategies to improve food environments across the United States [5, 7]. Between 2010 and 2012, for example, the Centers for Disease Control and Prevention (CDC)* Communities Putting Prevention to Work* (CPPW) program targeted health inequalities in several underserved communities in Los Angeles County. Through this funding, the Los Angeles County Department of Public Health implemented a number of nutrition interventions in the region. These interventions included (a) modifying food services and vending practices at food venues operated by county and city governments (e.g., incorporating healthy nutrition standards through the contracting process with food vendors or suppliers) [8]; (b) converting corner stores or other stores in low-income neighborhoods to food outlets which offer more fresh fruits and vegetables; and (c) utilizing outreach and health marketing to educate the public about the adverse effects of excess sugary drink consumption (Table 2) [9].

As in other communities, assuring community acceptance of SE modifications to the food environment requires in-depth knowledge and understanding of the key health behaviors and characteristics of targeted subpopulations [10]. To date, few ongoing public health strategies have tailored intervention programs to address these groups' unique needs. Access to more granular, community-level health data could change this practice by helping to better inform and guide planning and program improvements in these communities.

Capitalizing on the results from a local health and nutrition examination survey, we contribute to this gap in public health practice by studying a population that was exposed to and may have been affected by these and other nutrition interventions implemented in urban Los Angeles County during 2010–2012. The study examined local health data including predictors of healthy eating among a subset of low-income adults who receive free/low-cost services from multipurpose, public health centers in the jurisdiction. Policy and practice implications are discussed within the context of program improvement and future obesity prevention planning for the region.

## 2. Methods

Data from the first round of a local health and nutrition examination survey in Los Angeles County was collected during the first 15 months of the CPPW obesity prevention program. The survey included a subset of adults residing in low-income neighborhoods (verified using residential zip codes). Information collected by the survey included (a) objectively measured height and weight; (b) objectively measured waist circumference and blood pressure; (c) self-reported smoking status; (d) self-reported dietary behaviors; (e) ratings of self-efficacy in healthy eating and exercise; and (f) sociodemographics.

### 2.1. Survey Catchment Area

**Figure 1:**
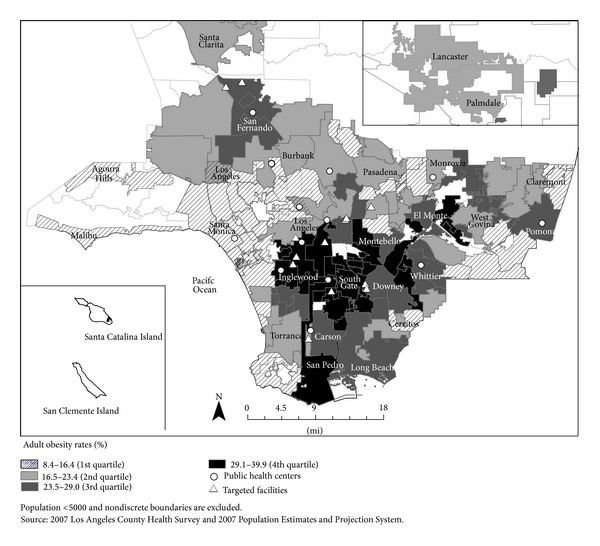
Public health center locations in relation to adult obesity burden in cities and communities of Los Angeles County, 2010–2012.

**Figure 2:**
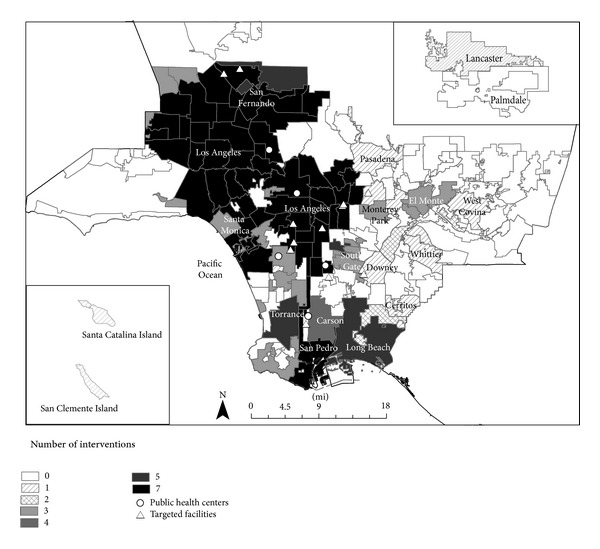
Public health center locations in relation to local obesity prevention interventions focused on nutrition, Los Angeles County, 2010–2012.

Survey participants in the subset were recruited from five out of the 14 low-income, multipurpose public health centers operated by the Los Angeles County Department of Public Health (LACDPH). Although services such as immunizations and treatment for sexually transmitted diseases were standard across all public health centers, not all community programming and outreach activities were the same. The five sites that were selected, for example, were located in regions with the highest economic hardship indices and the highest prevalence of adult obesity (Figure 1). In addition, the clients of these sites were among the intended audiences of several local obesity prevention efforts during 2010–2012.  Figure 2 shows selected center locations in relation to the nutrition interventions that were implemented by the CPPW program and other state or locally funded efforts.

### 2.2. Survey Population and Participant Recruitment

Survey participants were recruited by trained LACDPH staff in the waiting rooms of the five public health centers described above. LACDPH staff utilized a set of multistage, systematic procedures to recruit and enroll eligible participants during prespecified days of the survey period. These procedures accounted for such operational factors (when feasible) as each center's seasonal and daily clientele volume; time of day; types of services offered or programming provided; and clinic flow during the days of recruitment. All data collection activities took place between February and April, 2011.

### 2.3. Participant Eligibility and Informed Consent

To be eligible for the survey, participants had to: (1) be receiving services from the clinic during the recruitment period; (2) be at least 18 years of age; (3) be a resident of Los Angeles County; (4) not be pregnant; (5) speak English or Spanish; and (6) agree to complete a series of anthropometric and self-administered assessments on a specified scheduled weekend day in one of the designated health center locations. New and repeat center clients were equally recruited to participate. All prospective participants were asked for their names and dates of birth during eligibility screening; this information was monitored throughout the survey period to prevent individuals from participating more than once in the survey. As an incentive to participate, each participant was given a $50 gift card at the completion of the survey.

Informed consent was obtained from each participant prior to enrollment. Prior to fieldwork, all survey protocols and materials were reviewed and approved by the LACDPH Institutional Review Board.

### 2.4. Data Collection

Trained LACDPH staff including clinical personnel (e.g., public health nurses) measured heights and weights two to three times using a stadiometer (Seca 213) and a digital scale (Seca 876), respectively. Blood pressure (BP) measurements were measured using an automated sphygmomanometer and an appropriately sized cuff (Omron HEM-907XL). The final recorded height, weight, and BP measurements were the average of the repeated measurements. Each survey participant completed a standardized, self-administered questionnaire which included questions on sociodemographics, tobacco use, eating behaviors, and confidence about making changes to their diet and exercise routines. The seven-page paper questionnaire (available in both English and Spanish) was developed using previously validated questions from population health surveys in the literature, including the National Health and Nutrition Examination Survey (NHANES) [4] and the Los Angeles County Health Survey [2]. The diet questions, which asked about self-efficacy in healthy eating and exercise, were adapted from the validated Self-Efficacy for Diet and Exercise scale developed by Sallis and colleagues [12]. These questions (based on a 5-point Likert ranging from “I know I can” to “I know I cannot”) included “how sure are you that you can (a) …stick to low-fat foods when you feel depressed, bored, or tense; (b)… stick to low-fat foods when there is high fat food readily available at a party; (c) …stick to low-fat foods when dining with friends or co-workers; (d) …cut down on the amount of food you eat at each meal (to decrease portion size); and (e) …regularly read the serving size information listed on the Nutrition Facts label of packaged foods you eat.” The English version of the questionnaire was translated to Spanish using a standardized, forward-backward language translation protocol.

### 2.5. Statistical Analysis

Descriptive and univariate analyses were first performed to generate frequency distributions and standard statistics for each variable. Dependent and independent variables were identified, reviewed, and converted or transformed (as needed) to align with the statistical requirements of the various analyses. To assess overweight and obesity, we converted measured height and weight to body mass index (BMI = weight [kg]/height squared [m^2^]) using cut-off points for overweight and obese categories as defined by the CDC guidelines: BMI < 24.9, normal or nonobese; 25.0–29.9, overweight; ≥30.0, obese [13]. To assess prehypertension and hypertension ranges, diagnostic categories of blood pressure readings were created based on criteria recommended by the Joint National Committee on Prevention, Detection, Evaluation, and Treatment of High Blood Pressure, Seventh Report [*JNC 7*] [14]: systolic blood pressure (SBP) < 120 and diastolic blood pressure (DBP) < 80 = normal; SBP 120–139 or DBP 80–89 = prehypertension; SBP 140–159 or DBP 90–99 = stage 1 hypertension; SBP > 160 or DBP > 100 = stage 2 hypertension. To facilitate comparisons of eating behaviors within the subset of low-income adults, key dependent variables including fruit and vegetable consumption (e.g., ≥4 servings per day versus ≤3 servings per day) were dichotomized as* proxy* indicators of healthy eating. The analysis of cut-offs for the number of servings consumed was based on research evidence suggesting worse cardiovascular health outcomes for adults who consumed 3 or less servings of fruits and vegetables per day as compared to adults who consumed 3 or more fruits and/or 5 or more vegetables per day; this is in recognition that the recommended daily intake for any individual is generally based on age, gender, and physical activity level [15–17].

**Figure 3:**
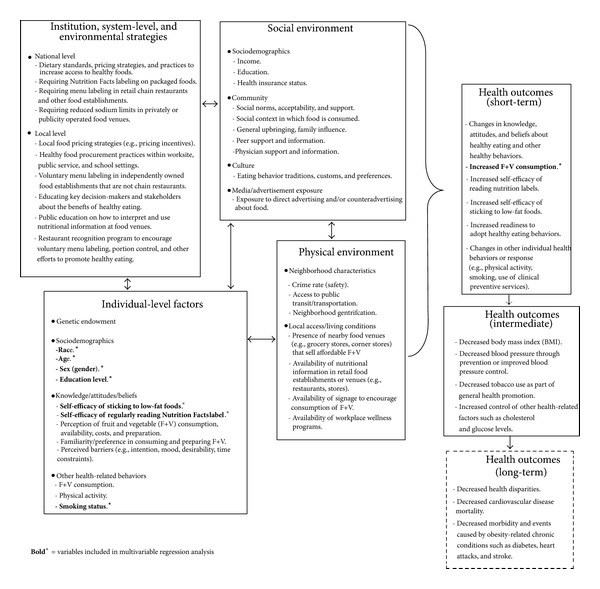
Logic framework: a socioecological perspective on healthy eating.

Where appropriate, Mantel-Haenszel chi-square tests and logistic regression procedures were performed to explore the relationships between participant characteristics (e.g., age, gender, education, employment, BMI, blood pressure, and smoking status) and participant behaviors (e.g., self-reported eating behaviors and self-efficacy in various aspects of healthy eating). Logistic regression analyses, adjusted for age and gender, were conducted to compare key indicators by race/ethnicity. Using consumption of ≥4 servings of fruit and vegetable as a* proxy* dependent variable for healthy eating, a series of multivariable regression models, adjusting for a range of covariates that are known to affect consumption of these foods [5, 18, 19], were constructed. These covariates included race, age, gender, education, BMI, blood pressure, smoking, and self-efficacy ratings on reading Nutrition Facts labels on the back of food packages. Variable inclusion in the models was guided by a logic framework based on the socioecological perspective (Figure 3) [5]. Selection(s) of the “self-efficacy” variable(s) for inclusion in each of the models were also informed by the results of bivariate analyses. Model 1, for example, explored the predictive associations between sociodemographics and fruit and vegetable consumption. Model 2 explored the predictive associations between cardiovascular disease risk factors and fruit and vegetable consumption. And model 3 explored the predictive associations between self-efficacy in healthy eating and fruit and vegetable consumption. In all models, fit was assessed using the Hosmer-Lemeshow Goodness-of-Fit test (*P* > 0.05). The final model was a synthesis of this iterative model building process. All data analyses were carried out using the SAS version 9.2 statistical software (SAS Institute Inc., Cary, North Carolina).

## 3. Results

Of the 1,393 prospective survey participants approached, 983 met eligibility criteria and were scheduled appointments. Of these, a total of 720 were low-income adults and completed the survey for a response rate of 74% for the subset. A large proportion of participants were black (40%) or Latino (34%), between the ages of 25 and 44 years (48%), and women (57%). More than one-third had a high school education or less (39%), nearly one-quarter were college graduates (22%), and over one-half were unemployed or underemployed (58%). Approximately two-thirds (68%) of the participants were overweight and/or obese; 30% were in the prehypertension range based on objectively measured blood pressure readings (Table 3). Although only 28% were reported to be current smokers, approximately 63% indicated exposure to second-hand smoke. In general, fruit and vegetable consumption was relatively low in the group, with only about one-fourth consuming four or more servings of fruits and/or vegetables per day (26%).

In the comparison analysis (see Table 4), Latinos were more likely than whites to be overweight and obese (adjusted odds ratio [AOR] = 3.9, 95% confidence interval [CI] = 2.2, 6.9). Similarly, blacks were more likely than whites to be overweight and obese (AOR = 2.1, 95% CI = 1.2, 3.5). Latinos were generally less likely to smoke, as compared to whites (AOR = 0.4, 95% CI = 0.2, 0.7). Based on objectively measured blood pressures, Latinos and blacks experienced a greater burden of elevated blood pressure readings than whites: 49% of Latinos (AOR = 1.2, 95% CI = 0.7, 2.0) and 55% of blacks (AOR = 1.4, 95% CI = 0.8, 2.4) had readings in the prehypertension and hypertension ranges.

In multivariable regression analyses (see Table 5), being a woman and having a high self-efficacy for regularly reading Nutrition Facts labels were strong predictors of high fruit and vegetable consumption. In the final model, women were 1.5 times (95% CI = 1.0, 2.1) more likely than men to consume 4+ servings of fruits and vegetables per day. Participants with high self-efficacy in reading Nutrition Facts labels were 2.4 times (95% CI = 1.7, 3.5) more likely than their counterparts (with low self-efficacy) to do the same. The Hosmer and Lemeshow Goodness-of-Fit test indicated that these models were compatible with the data presented (*χ* ^2^ = 5.57, *P* = .70).

## 4. Discussion

Guided by a socioecological framework [5], the present study conducted a series of analyses to examine key characteristics of a subpopulation disproportionately affected by overweight and obesity in Los Angeles County [1]. This priority group is one of several vulnerable groups targeted by a number of nutrition-focused obesity prevention interventions in the region (Table 1) [7, 8]. Although prior efforts have relied on national and/or county surveillance databases to aid program planning [1, 2, 4, 10, 20], this study is among the first to collect more granular, community-level health data that are representative of the groups targeted by program interventions that sought to make changes to the food environment. These data have implications for quality improvement, especially for local health authorities and community-based organizations seeking to improve or better tailor program delivery to their intended audiences [21].

While emerging evidence supports the use of system and environmental change strategies [5, 7, 22], there remains a paucity of research that has fully elucidated the interactions between these structural modifications and individual health behavior change. To achieve meaningful outcomes in community and individual health, interventions often require substantive tailoring to match the needs and the unique social, epidemiological, and ecological characteristics of the target subpopulations [21, 23]. Oka and colleagues (2013), for instance, analyzed a community-based epidemiologic survey using multilevel modeling to better understand area-based variations in obesity [24]. They demonstrated differences in obesity prevalence by gender and race/ethnicity at the neighborhood level and concluded that, to be effective, future interventions/programs should address these and other neighborhood-specific characteristics.

In the present study, the sampled population had high prevalence of overweight and obesity; this was accentuated for Latinos and blacks. This high prevalence, however, is not uniquely different from the documented evidence in the literature for US minorities [6, 25]. In the literature, disparities in obesity burden, including associated conditions such as hypertension, generally clustered in vulnerable groups, frequently confounded by multiple social and environmental factors that are not solely explained by socioeconomic status [26]. These factors have included but are not limited to racism [27, 28], residential segregation [29], and the built environment [30].

The most striking finding in the study was that gender and self-efficacy were strong predictors of healthy eating (e.g., fruit and vegetable consumption), even after controlling for a number of confounding variables including other demographics in the sampled group. This was somewhat unexpected given that the subset of adults included in the analysis represented a source population believed to be ready for and would benefit from structural changes made to the food environment (e.g., healthy food procurement, corner store conversion, 100% healthy vending machine policy, and competitive pricing of healthy foods in food venues). However, experiential information suggests that due to perceived lower educational attainment and poor nutrition in this population, differential patterns of receptivity or readiness to capitalize on these changes were likely common. As such, after adjusting for covariates such as age, race, and education, survey participants were significantly more likely to consume fruits and vegetables than other participants when they were women and had higher self-efficacy in reading Nutrition Facts labels.

From a practical standpoint, intervening with women who typically make food selection decisions for their entire household (nutrition gatekeepers) may be advantageous to the overall effort to reduce obesity in Los Angeles County, as it can concurrently model positive changes in the diet of the entire family unit and can be applied across generations [31, 32]. Intervening with women can also indirectly target other members of the household, especially men who often eat poorly [33, 34]. The potentially additive effect that may result from this action could augment the structural changes (i.e., through system and environmental interventions) made to the food environment by the recent federal and local obesity prevention initiatives. Similarly, improving the level of confidence in practicing healthy eating behaviors may also help accelerate the community acceptance of changes made to the food environments by these efforts. To achieve optimal interventional effects, priming at-risk groups to accept and take advantage of structural improvements may be as important as improving the food environments themselves [10, 35].

The present study was subject to a number of limitations. First, generalizability of the findings to the general population in Los Angeles County was not feasible, as the sampled group principally represented the region's low-income adult population. This, however, should not be considered a study weakness, as the demographics of the group aligned closely with the intended audiences of the various federal and local obesity prevention efforts in the region. Second, large confidence intervals were observed for some of the point estimates in the analyses. For most of these, the smaller sample size(s) of the referent group (i.e., whites) likely contributed to the imprecision. Third, self-selection and self-reporting bias likely led survey participants to over- or underestimate their food frequency and reports of self-confidence (self-efficacy in healthy eating). Fourth, the study design was cross-sectional in nature and thus was only able to describe the regional health profiles of the subpopulation at one given point in time. Finally, measurement errors, including misclassifications, likely introduced additional bias to the descriptive and comparison analyses. These potential errors, however, were minimized through iterative use of well-defined, standardized measurement protocols and rigorous training of field staff responsible for data collection.

Despite these limitations, community-level health data on particular subpopulations in Los Angeles County highlight the feasibility and utility of collecting these kinds of data to address socioecological factors that drive healthy eating in urban settings. To increase desired eating behaviors, both structural (system or environmental change) and individual-level approaches (e.g., changes in knowledge, attitudes, beliefs, intentions, and self-efficacy) should be employed. Developing nutrition interventions tailored to the unique characteristics of targeted subpopulations can help prepare individuals to take advantage of the structural improvements or resources that are made available to them by obesity prevention initiatives. Such tailoring of structural change interventions can be synergistically augmented by culturally sensitive public education and/or community engagement that seeks to address the underlying gender norms and self-confidence mediators known to shape eating behaviors.

## 5. Conclusions

Although local health data can help facilitate community planning and acceptance of system-level and environmental changes to the food environment, area-specific health profiles and behavioral determinants of healthy eating in the targeted subpopulations are often not well-characterized prior to nutrition program implementation or for use in subsequent program improvement efforts. The present study addresses this gap in public health practice by providing actionable data that the LACDPH can use to further address health disparities in the region. Many of the lessons learned in Los Angeles County may have similar applications in other US communities. Local or community health assessments represent a set of tools that is often underutilized by public health authorities. Ultimately, these chronic disease surveillance and assessment tools that document more granular information about subpopulations' health status provide the opportunity for communities to tailor multisector public health programs to intended audiences.

## Figures and Tables

**Table 1:** Obesity prevalence among cities and communities in Los Angeles County, by economic hardship ranking, 2011.

Top 10 (most affluent)			Bottom 10 (lowest socioeconomic status)		
City/community	Obesity prevalence (%)	Rank, economic hardship (1–127)	City/community	Obesity prevalence (%)	Rank, economic hardship (1–127)
San Marino	8.4	1	East Compton	39.9	127
Marina Del Rey	9.9	2	Willowbrook	39.5	126
La Canada Flintridge	10.1	3	Compton	39.1	125
Beverly Hills	10.4	4	Florence-Graham	38.7	124
Malibu	10.4	4	Lynwood	37.8	123
Palos Verdes Estates	11.8	6	City of Los Angeles		
Rolling Hills Estates	11.9	7	Council District 9	36.7	122
Santa Monica	11.9	7	Paramount	35.5	121
South Pasadena	11.9	7	Westmont	35.4	120
Calabasas	12.3	10	City of Los Angeles		
			Council District 8	35.1	119
			West Athens	33.2	118

Average	11.0	—	Average	37.0	—

The economic hardship index is scored by combining six indicators: crowded housing, percentage of persons living below the federal poverty level, percentage of persons over the age of 16 years who are unemployed, percentage of persons over the age of 25 years without a high school education, dependency, and per capita income.

*Data source*: Office of Health Assessment and Epidemiology, Los Angeles County Department of Public Health [1].

**Table 2:** Summary of nutrition-focused, system and environmental change strategies in Los Angeles County, 2010–2012.

Type of strategy	Target setting	Strategy summary
Institutional policies or practices on healthy food procurement, for food and vending services.	Government	(i) County of Los Angeles Board motion mandating healthy nutrition standards and food procurement practices in 37+ county departments.
	Cities	(ii) Adoption of healthy nutrition standards and food procurement practices in at least ten low-income cities with high obesity prevalence.

Breastfeeding promotion and accommodations in the workplace.	Government Private employers	(i) Institutional policy to provide lactation accommodations in the workplace for county departments and other employers in the region.
	Hospitals	(ii) Attaining “Baby Friendly” hospital certifications to increase breastfeeding promotion at four to five large, safety-net hospitals in low-income areas of Los Angeles County.

Improving food quality in grocery stores, corner stores, and/or farmers markets.	Cities	Efforts to increase access to healthy foods through corner store conversions and farmers markets in at least two cities with low-income neighborhoods.

Public education through health marketing and other social media approaches.	County/city general population	Dissemination of multipronged public education campaigns (e.g., sodium and sugary drink reduction campaigns) designed to promote healthy eating in the community through social and traditional media channels.

**Table 3:** Sociodemographic characteristics and cardiovascular risk profiles of participants from the local health and nutrition examination survey, Los Angeles County, 2011.

Characteristics	*n* (%)
**T** **o** **t** **a** **l** ^a^	**720 (100)**
Sociodemographics	
Gender	
Women	408 (57)
Men	312 (43)
Age (years)	
18–24	150 (21)
25–44	346 (48)
45–64	203 (28)
65+	21 (3)
Race/ethnicity	
Black	288 (40)
Hispanic/Latino	241 (33)
White	77 (11)
Asian/Pacific Islander	72 (10)
Other	40 (6)
Education	
Less than high school	121 (17)
High school graduate	159 (22)
Some college or junior college	280 (39)
College graduate/postgraduate	155 (22)
Employment	
Employed^b^	231 (32)
Unemployed/underemployed^c^	417 (58)
Retired/disabled	65 (9)
Cardiovascular health	
Body mass index or BMI (measured)^d^	
Underweight	10 (1)
Normal	221 (31)
Overweight	229 (32)
Obese	259 (36)
Blood pressure, mm Hg (measured)^e^	
Normal	342 (48)
Prehypertension	213 (30)
Hypertension^f^	165 (23)
Diabetes (self-report)	
Diabetic^g^	53 (7)
Smoking (self-report)	
Current smoker	199 (28)
Exposed to second-hand smoke in the past 7 days	343 (48)

^a^Data collection was carried out at five designated public health centers during the survey period, February–April 2011. Percentage and number of cases may not add up to 100% or to the total due to rounding and missing information.

^b^Employed: employed full-time or self-employed.

^c^Underemployed: employed part-time.

^d^Based on the Centers for Disease Control and Prevention (CDC) guidelines for body mass index (BMI) calculations: BMI = weight (kg)/height (m^2^); BMI classifications = BMI ≤ 24.9 (normal or nonobese), BMI 25.0–29.9 (overweight), and BMI ≥ 30.0 (obese).

^e^Based on classifications [11]: normal blood pressure (systolic < 120 mm Hg and diastolic < 80 mm Hg); prehypertension (systolic 120–139 mm Hg or diastolic 80–89 mm Hg); hypertension (stage 1, systolic 140–159 mm Hg or diastolic 90–99 mm Hg, and stage 2, systolic > 160 mm Hg or diastolic > 100 mm Hg).

^f^Included participants with controlled (on medication) and uncontrolled stage 1 or stage 2 hypertension. Example: participants who were on medication(s) but have readings in the normal or prehypertension range were classified as having “controlled” or “uncontrolled” hypertension.

^g^Diabetic: have been told by a doctor they have diabetes and/or were taking diabetes medication(s) as verified by the medication list collected during the survey.

**Table 4:** Health indicators and eating behaviors of survey participants by race/ethnicity, Los Angeles County, 2011.^a^

	White (referent) *n* (%)	Latino			Black			Asian/PI		
		*n* (%)	Crude odds ratio COR (95% CI)	Adjusted odds ratio AOR (95% CI)	*n* (%)	Crude odds ratio COR (95% CI)	Adjusted odds ratio AOR (95% CI)	*n* (%)	Crude odds ratio COR (95% CI)	Adjusted odds ratio AOR (95% CI)
**Total**	**77 (11)**	**241 (36)**			**287 (42)**			**72 (11)**		
Health indicators										
BMI (measured)^b^										
Overweight and obese	40 (52)	190 (79)	3.45 (2.00, 5.93)***	3.93 (2.24, 6.91)***	197 (69)	2.03 (1.21, 3.38)**	2.08 (1.22, 3.54)**	36 (50)	0.93 (0.49, 1.76)	0.95 (0.49, 1.85)
Blood pressure, mm Hg (measured)^c^										
Normal	41 (53)	122 (50)	0.90 (0.54, 1.51)	0.86 (0.50, 1.48)	129 (45)	0.71 (0.43, 1.18)	0.71 (0.41, 1.21)	45 (63)	1.46 (0.76, 2.82)	1.5 (0.74, 3.09)
Prehypertension or hypertension (stage 1 or 2)	36 (47)	119 (49)	1.11 (0.66, 1.86)	1.17 (0.67, 2.02)	159 (55)	1.40 (0.85, 2.32)	1.42 (0.82, 2.43)	27 (38)	0.68 (0.36, 1.32)	0.66 (0.32, 1.34)
Smoking (self-report)										
Current tobacco user	28 (36)	42 (17)	0.37 (0.21, 0.65)**	0.39 (0.22, 0.70)**	104 (36)	0.99 (0.59, 1.67)	1.18 (0.69, 2.02)	13 (18)	0.39 (0.18, 0.82)*	0.44 (0.20, 0.96)*
Exposed to second-hand smoke^d^	54 (74)	138 (72)	0.90 (0.49, 1.66)	0.92 (0.50, 1.70)	205 (79)	1.36 (0.74, 2.49)	1.42 (0.77, 2.49)	49 (75)	1.08 (0.50, 2.33)	1.11 (0.52, 2.41)
Chronic diseases/conditions (self-report)										
Diagnosed with diabetes and/or were on diabetes medication(s)	5 (6)	17 (7)	1.09 (0.39, 3.07)	1.05 (0.36, 3.01)	23 (8)	1.25 (0.46, 3.40)	1.03 (0.37, 2.90)	6 (8)	1.31 (0.38, 4.49)	1.26 (0.35, 4.51)
Self-reported eating behaviors										
Fruit and vegetable consumption										
Consumed fruits and/or vegetables 4+ per day	17 (22)	59 (25)	1.15 (0.62, 2.13)	1.10 (0.59, 2.05)	77 (27)	1.29 (0.71, 2.34)	1.19 (0.65, 2.17)	16 (22)	1.01 (0.47, 2.19)	0.93 (0.42, 2.02)
Reported drinking the following two or more times per day										
Carbonated beverages (regular soda)	12 (16)	31 (13)	0.80 (0.39, 1.65)	0.81 (0.40, 1.68)	44 (15)	0.99 (0.49, 1.98)	1.09 (0.54, 2.21)	4 (6)	0.32 (0.10, 1.04)	0.35 (0.11, 1.14)
100% fruit juice	15 (19)	52 (22)	1.14 (0.60, 2.17)	1.13 (0.59, 2.15)	82 (28)	1.65 (0.89, 3.06)	1.68 (0.90, 3.14)	11 (15)	0.75 (0.32, 1.75)	0.75 (0.32, 1.76)
Other sugar-sweetened beverages	15 (20)	29 (12)	0.57 (0.29, 1.23)	0.57 (0.29, 1.14)	39 (14)	0.65 (0.34, 1.25)	0.65 (0.34, 1.27)	8 (11)	0.52 (0.21, 1.31)	0.53 (0.21, 1.35)
Reported low confidence in sticking to low-fat foods when										
Feeling depressed, bored, or tense	41 (54)	111 (47)	0.75 (0.45, 1.26)	0.72 (0.43, 1.22)	149 (52)	0.92 (0.56, 1.53)	0.88 (0.53, 1.48)	35 (49)	0.81 (0.42, 1.54)	0.78 (0.41, 1.49)
High-fat foods are readily available at a party	49 (64)	151 (63)	0.97 (0.57, 1.65)	0.95 (0.55, 1.62)	163 (57)	0.76 (0.45, 1.27)	0.75 (0.44, 1.28)	48 (68)	1.19 (0.60, 2.35)	1.25 (0.63, 2.49)
Dining with friends or coworkers	40 (52)	131 (55)	1.14 (0.68, 1.91)	1.15 (0.68, 1.94)	146 (52)	0.99 (0.60, 1.63)	1.03 (0.62, 1.71)	46 (66)	1.77 (0.91, 3.45)	1.87 (0.95, 3.66)
Portion size control—reported low confidence in										
Cutting down the food eaten in each meal	22 (29)	78 (33)	1.20 (0.68, 2.11)	1.27 (0.71, 2.25)	114 (40)	1.62 (0.93, 2.80)	1.83 (1.04, 3.22)*	27 (38)	1.51 (0.76, 3.00)	1.66 (0.82, 3.36)
Reading serving size information on labels	24 (31)	103 (43)	1.67 (0.97, 2.89)	1.80 (1.03, 3.14)*	126 (44)	1.74 (1.02, 2.97)*	1.93 (1.11, 3.33)*	34 (47)	1.98 (1.01, 3.85)*	2.23 (1.13, 4.41)*

^a^Data collection was carried out at five designated public health centers during the survey period, February–April 2011. Percentage and number of cases may not add up to 100% or to the total due to rounding and missing information. Models were stratified by race/ethnicity and adjusted for age and gender.

^b^Based on the Centers for Disease Control and Prevention (CDC) guidelines for body mass index (BMI) calculations: BMI = weight (kg)/height (m^2^); BMI classifications = BMI ≤ 24.9 (normal or nonobese), BMI 25.0–29.9 (overweight), BMI ≥ 30.0 (obese).

^c^Based on classifications [11] normal blood pressure (systolic < 120 mm Hg and diastolic < 80 mm Hg); prehypertension (systolic 120–139 mm Hg or diastolic 80–89 mm Hg); hypertension (stage 1, systolic 140–159 mm Hg or diastolic 90–99 mm Hg, and stage 2, systolic > 160 mm Hg or diastolic > 100 mm Hg).

^d^Exposed “within the past 7 days.”

*P < 0.05, **P < 0.01, ***P < 0.001.

**Table 5:** Predictors of high fruit and vegetable consumption (4+ servings per day) among survey participants, Los Angeles County, 2011.

Independent variables	Model 1^a^	Model 2^b^	Model 3^c^	Full model^d^
	Adjusted odds ratio (95% CI)	Adjusted odds ratio (95% CI)	Adjusted odds ratio (95% CI)	Adjusted odds ratio (95% CI)
Sociodemographic				
Gender (women versus men)	1.65 (1.16, 2.341)**	1.66 (1.16, 2.39)**	1.46 (1.01, 2.13)*	1.48 (1.02, 2.14)*
Age (18–49 years versus 50+ years)	0.87 (0.59, 1.28)	0.88 (0.59, 1.33)	0.88 (0.58, 1.33)	0.87 (0.58, 1.32)
Race (black versus white)	1.16 (0.63, 2.15)	1.21 (0.65, 2.26)	1.28 (0.68, 2.44)	1.30 (0.70, 2.44)
Race (Latino versus white)	1.07 (0.56, 2.02)	1.10 (0.57, 2.12)	1.15 (0.59, 2.25)	1.18 (0.61, 2.30)
Education Level (greater than high school versus less than high school education)	0.87 (0.61, 1.26)	1.19 (0.82, 1.72)	1.32 (0.89, 1.94)	0.75 (0.52, 1.10)
Cardiovascular health				
Body mass index (BMI) (normal/nonobese versus overweight and obese)	—	1.42 (0.96, 2.10)	—	1.38 (0.93, 2.04)
Blood pressure status (normal versus prehypertension/hypertension)	—	0.80 (0.55, 1.67)	—	0.75 (0.51, 1.10)
Smoking status (nonsmoker versus smoker)	—	0.78 (0.52, 1.17)	—	0.82 (0.54, 1.25)
Self-efficacy for engaging in healthy eating				
Reading serving size information listed on Nutrition Facts label of packaged foods (high versus low confidence level)^e^	—	—	2.15 (1.35, 3.41)**	2.39 (1.67, 3.50)***
Reducing portion sizes at each meal (high versus low confidence level)^e^	—	—	1.04 (0.65, 1.69)	—
Sticking to low-fat foods when depressed, bored, or tense (high versus low confidence level)^e^	—	—	1.19 (0.75, 1.88)	—
Sticking to low-fat foods when high-fat foods available at a party (high versus low confidence level)^e^	—	—	1.22 (0.74, 2.03)	—
Sticking to low-fat foods when dining with friends or coworkers (high versus low confidence level)^e^	—	—	1.23 (0.76, 2.00)	—

CI: confidence interval; *χ* ^2^_HL_: *Hosmer-Lemeshow* chi-square test.

^a^Model 1 adjusted for gender, age, race, and education level; *χ* ^2^_HL_ = 4.37, *P* = 0.82.

^b^Model 2 adjusted for gender, age, race, BMI, blood pressure status, and smoking status; *χ* ^2^_HL_ = 6.31, *P* = 0.61.

^c^Model 3 adjusted for gender, age, race, education level, and self-efficacy for reading serving size information on Nutrition Facts labels, reducing portion sizes, and sticking to low-fat foods (i.e., self-efficacy variables were dichotomized using level of confidence data reported on a 1–5 Likert scale); *χ* ^2^_HL_ = 6.66, *P* = 0.57.

^d^Full model comprises the statistically significant variables and/or other relevant covariates included in models 1–3; *χ* ^2^_HL_ = 5.57, *P* = 0.70.

^e^Measure of confidence level based on a 5-point Likert scale “I know I can” to “I know I cannot.”

*P < 0.05, **P < 0.01, and ^∗∗∗^ *P* < 0.001.
